# Macrophage colony-stimulating factor and its receptor signaling augment glycated albumin-induced retinal microglial inflammation in vitro

**DOI:** 10.1186/1471-2121-12-5

**Published:** 2011-01-25

**Authors:** Wei Liu, Ge Z Xu, Chun H Jiang, Jie Tian

**Affiliations:** 1 Department of Opthalmology, EENT Hospital, Eye Institute, Fudan University, Shanghai, 200031, China; 2 Institute of Brain Science, Fudan University, Shanghai, 200031, China

## Abstract

**Background:**

Microglial activation and the proinflammatory response are controlled by a complex regulatory network. Among the various candidates, macrophage colony-stimulating factor (M-CSF) is considered an important cytokine. The up-regulation of M-CSF and its receptor CSF-1R has been reported in brain disease, as well as in diabetic complications; however, the mechanism is unclear. An elevated level of glycated albumin (GA) is a characteristic of diabetes; thus, it may be involved in monocyte/macrophage-associated diabetic complications.

**Results:**

The basal level of expression of M-CSF/CSF-1R was examined in retinal microglial cells *in vitro*. Immunofluorescence, real-time PCR, immunoprecipitation, and Western blot analyses revealed the up-regulation of CSF-1R in GA-treated microglial cells. We also detected increased expression and release of M-CSF, suggesting that the cytokine is produced by activated microglia via autocrine signaling. Using an enzyme-linked immunosorbent assay, we found that GA affects microglial activation by stimulating the release of tumor necrosis factor-α and interleukin-1β. Furthermore, the neutralization of M-CSF or CSF-1R with antibodies suppressed the proinflammatory response. Conversely, this proinflammatory response was augmented by the administration of M-CSF.

**Conclusions:**

We conclude that GA induces microglial activation via the release of proinflammatory cytokines, which may contribute to the inflammatory pathogenesis of diabetic retinopathy. The increased microglial expression of M-CSF/CSF-1R not only is a response to microglial activation in diabetic retinopathy but also augments the microglial inflammation responsible for the diabetic microenvironment.

## Background

Recent evidence strongly suggests that microglial activation plays a central role in the inflammation induced by experimental and human retinopathy [1-4]. Microglia, the resident macrophages of the central nervous system (CNS), is sensitive to minute changes in their microenvironment and is quickly activated. Upon activation, they proliferate and become amoeboid phagocytotic cells that produce a variety of proinflammatory cytokines, nitric oxide (NO), and reactive oxygen intermediates [5-7]. These factors are well known to induce neurodegeneration, although the precise mechanism is not fully understood.

Recently, Wang et al. [8] reported that glycated albumin (GA) significantly enhanced the production and release of tumor necrosis factor-α (TNF-α) from retinal microglia *in vitro*, suggesting that GA contributes to microglial inflammation in diabetic retinopathy. Chronic hyperglycemia in diabetes, through the nonenzymatic glycation of free amino groups in proteins by glucose, leads to the formation of labile Schiff base intermediates that undergo Amadori rearrangement, leading to the relatively stable early adducts ketoamine or fructosamine (so-called Amadori products). Eventually, these Amadori products form irreversible advanced glycation end products (AGEs) [9]. Glycated albumin levels increase drastically under diabetic conditions, and the plasma levels of GA may vary from normal (400 μg/mL) to diabetic (1000 μg/mL) [10]. Increasing evidence suggests that early glycated albumin is not just an index of glycemia or the precursor of AGEs. By itself, it may have direct impacts on cellular functions and thus may play a pathophysiological role in microvascular complications of diabetic nephropathy and retinopathy [11-14]. Glycated albumin accumulates in the diabetic retina [14-16] and changes the local concentrations of cytokines, growth factors, and other bioactive molecules by binding on several cell types, such as retinal pigment epithelium cells [17,18] and monocytes/macrophages [19,20], and by inducing the secretion of proinflammatory cytokines via the activation of protein kinase C (PKC), nuclear factor-κB (NF-κB), protein tyrosine kinase (PTK), and activator protein-1 (AP-1) signaling [21,22]. Therefore, GA may have important effects on the initiation and progression of diabetic retinopathy.

Macrophage colony-stimulating factor (M-CSF) is one of the most important substances known to affect macrophage physiology. The binding of M-CSF to its sole specific receptor, CSF-1R, stimulates the survival, proliferation, and differentiation of mononuclear phagocytes [23,24]. Moreover, M-CSF is considered a key cytokine in the regulation of microglial inflammatory responses [25]. Accumulating evidence suggests the up-regulation of M-CSF accompanied by the strong and selective induction of CSF-1R in activated microglia following brain damage caused by injury or disease such as brain ischemia or Alzheimer's disease [26-29]. *In vitro*, microglial overexpression of CSF-1R augments phagocytosis and contributes to the inflammatory response [30,31]. Similar patterns of M-CSF/CSF-1R expression have also been reported in the diabetic environment, suggesting that M-CSF/CSF-1R signaling plays a critical role in the pathogenesis of diabetic lesions [32,33]. The exact mechanism, however, is unclear.

Given the small amount of information available concerning CSF-1R expression by microglia in the diabetic environment, the regulatory role of M-CSF/CSF-1R signaling in microglial inflammation in diabetic retinopathy is unknown. In the present study, we sought to ascertain whether GA has an effect on retinal microglial activation, including the production of proinflammatory cytokines, as well as the expression of M-CSF and its receptor CSF-1R. Furthermore, using exogenous M-CSF and antibody neutralization, we assessed the combined effect of M-CSF and GA on the proinflammatory response in primary microglial cells. Our results indicate that M-CSF acts as a costimulatory molecule to synergize GA-induced microglial inflammation via binding to its overexpressed receptor CSF-1R in diabetic retinopathy.

## Results

### Morphology and characterization of cultured newborn rat microglia

After 7 to 8 days of mixed-cell culture, the microglia appeared in small colonies (about 2 to 5 cells) of highly refractive cells above the basal macroglial layer. These round microglial cells or colonies were loosely attached to the macroglial monolayer; maximum proliferation was observed within 10 to 12 days. The purified microglial cells went from being amoeboid in shape to having a ramified shape, with a single long process and a small cell soma, over the course of 2 to 3 days. Immunofluorescence analysis revealed that OX-42-positive microglial cells accounted for 95% of the harvested cells, whereas none of the cells was positive for GFAP.

### Effects of various agents on microglial viability by MTT assay

**Figure 1:**
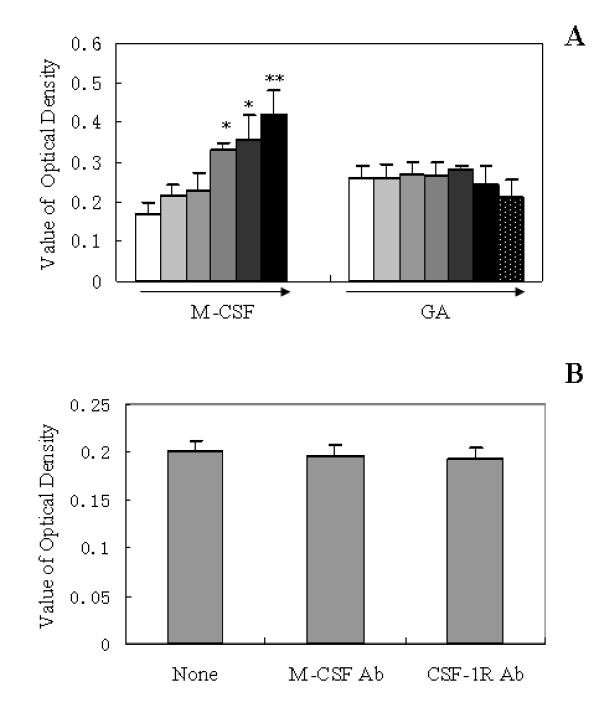
**Effect of M-CSF, glycated albumin (GA) and the antibodies of either M-CSF(anti-M-CSF Ab)or CSF-1R(anti-CSF-1R Ab) on microglial viability**. After incubated with various stimuli such as M-CSF or GA (A), and anti-M-CSF Ab (1 μg/ml) or anti-CSF-1R Ab (1 μg/ml) (B) for 24 h, the cell viability was assessed by MTT assay. The gradient concentrations of stimuli were: 5, 10, 25, 50, 100 ng/ml for M-CSF; and 10, 50, 100, 500, 1000, 2000 μg/ml for GA. The results are shown as means ± SD of three individual experiments. Only M-CSF induced microglial proliferation in a dose-dependent manner, other agents have no effects on microglial viability. One-way ANOVA was performed, followed by post-hoc analysis. *, P < 0.05; **, P < 0.005;

M-CSF-induced microglial proliferation in a dose-dependent manner was confirmed, in which the extent of microglial proliferation correlated well with the increase of the viability measured by MTT assay. In contrast to M-CSF, GA didn't induce significant proliferation (Figure 1A). Furthermore, the microglial apoptosis was observed when the concentration of GA exceeds 1000 μg/mL. In addition, the antibodies of either M-CSF or CSF-1R have no effect on microglial viability by MTT assay (Figure 1B).

### GA causes morphological changes in microglial cells

**Figure 2:**
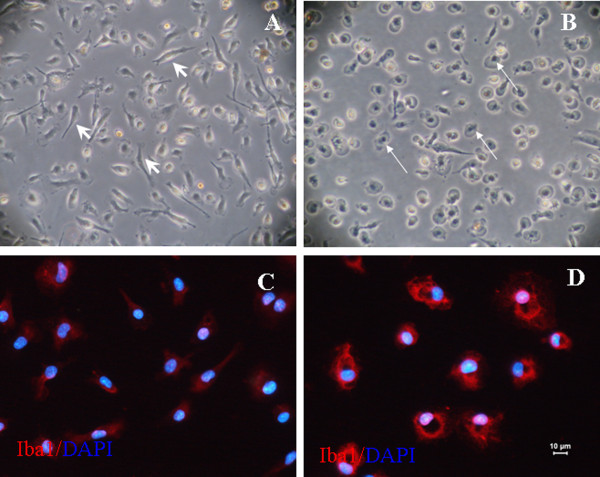
**Effect of glycated albumin (GA) on cultured retinal microglial cells**. (A) Phase-contrast image of purified retinal microglial cells after 72 h. The cells have a ramified shape, with a single long process and a small cell body (short arrow). (B) Following exposure to 250 μg/mL GA for 24 h, the cells became rounder, larger, and amoeboid in shape (long arrow) (original magnification: × 100). (C) Microglial cells without GA exposure show weak Iba1 immunofluorescence (red) in the cytoplasm. (D) Following exposure to 250 μg/mL GA for 24 h, Iba1 expression was up-regulated, and the protein was translocated to membrane ruffles. The nuclei of the microglia are labeled with DAPI (blue).

As shown in Figure 2, the unstimulated microglia had a ramified shape *in vitro *(Figure 2A). In contrast, the GA-stimulated microglia was bigger and rounder, with the appearance of activated cells (Figure 2B).

### Effect of GA on the expression of microglial Iba1

Iba1, a microglia-specific calcium-binding protein and marker of activated microglia, is involved in motility, phagocytosis, and proinflammation in activated microglia via the regulation of the actin cytoskeleton [34]. In this study, we observed enhanced Iba1 expression in GA-stimulated microglia compared with unstimulated cells. In addition, following 24 h of stimulation with GA, Iba1 was translocated from the cytoplasm to membrane ruffles (Figure 2C, 2D).

### GA-induced release of TNF-α and interleukin (IL)-1β from activated microglia

**Figure 3:**
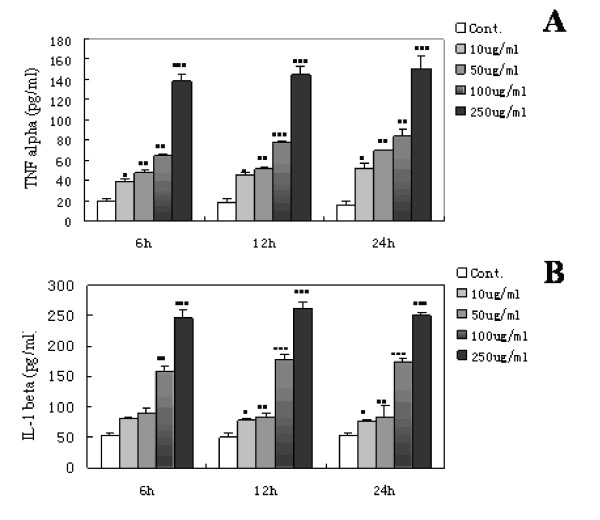
**Cytokine release from purified microglial cells stimulated with glycated albumin (GA) for 6, 12, or 24 h**. (A) The release of TNF-α from retinal microglial cells cultured with 0, 10, 50, 100, or 250 μg/mL GA for various time periods. TNF-α in the culture supernatants began to increase at 6 h after exposure to 10 μg/mL GA and peaked after exposure to 250 μg/mL GA. (B) The release of IL-1β from retinal microglial cells cultured with 0, 10, 50, 100, or 250 μg/mL GA for various time periods. IL-1β in the culture supernatants began to increase at 12 h after exposure to 10 μg/mL GA and peaked after exposure to 250 μg/mL GA. The results are shown as means ± SD of three individual experiments. One-way ANOVA was performed, followed by post-hoc analysis. *, P < 0.05; **, P < 0.001; ***, P < 0.0001.

**Figure 4:**
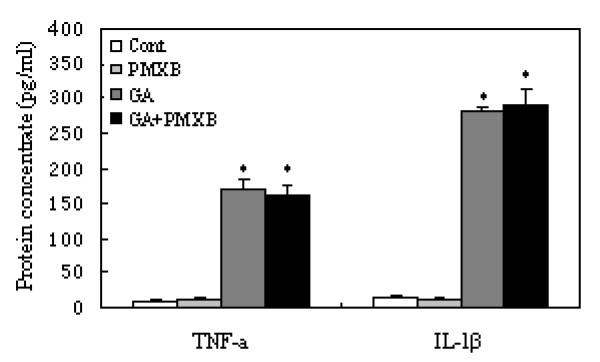
**Effect of the endotoxin-binding polypeptide polymyxin B (PMXB) on cytokine production by microglia stimulated with glycated albumin (GA) for 24 h**. Neither TNF-α nor IL-1β production induced by 250 μg/mL GA was inhibited by PMXB (10 μg/mL). Unstimulated microglia was as a control. The results are shown as means ± SD of three individual experiments. One-way ANOVA was performed, followed by post-hoc analysis. *, P < 0.0001

Following stimulation with 10 μg/mL GA for 6 h, a significant increase in the release of TNF-α was detected compared with the control group (38.7 ± 3.2 vs. 19.7 ± 1.7pg/mL; P < 0.05). Moreover, GA induced the dose-dependent release of TNF-α, with a peak at 250 μg/mL (138.2 ± 6.6pg/mL; P < 0.0001). There was a slight increase in the release of TNF-α from 6 to 24 h; however, no statistically significant difference was found among the various groups (Figure 3A). Similarly, GA induced the dose-dependent release of IL-1β. Different from the release of TNF-α, the increase of IL-1β following stimulation with 10 μg/mL GA was not significant compared with that in the control group until after 12 h (78.1 ± 3 vs. 50.8 ± 6.8pg/mL; P < 0.05). The release of IL-1β peaked at 261.3 ± 11.4pg/mL following 12 h of stimulation with 250 μg/mL GA (P < 0.0001); however, no statistically significant difference was found among the various groups (Figure 3B). Neither TNF-α nor IL-1β production by activated microglia stimulated with GA could be prevented by the endotoxin binding polypeptide polymyxin B (PMXB) at 10 μg/mL, a dosage previously reported to abrogate LPS-induced effects on microglia [35] (Figure 4).

### GA-induced production of M-CSF by activated microglia

**Figure 5:**
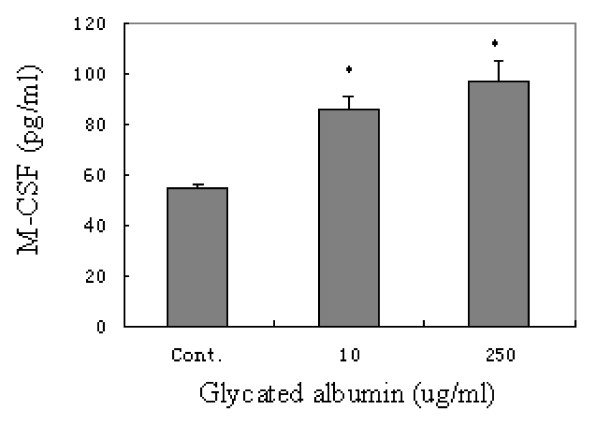
**M-CSF release from purified microglial cells stimulated with 0, 10, or 250 μg/mL glycated albumin (GA) for 24 h**. GA significantly induces the release of M-CSF, compared with the control. The results are shown as means ± SD of three individual experiments. One-way ANOVA was performed, followed by post-hoc analysis. *, P < 0.05.

M-CSF production occurs via autocrine signaling in activated microglia. To determine whether GA could induce microglial production of M-CSF, we analyzed the amount of M-CSF in the microglial culture medium. After stimulation with 10 or 250 μg/mL GA for 24 h, a significant increase in M-CSF release (86.4 ± 4.8 or 97.4 ± 7.3 pg/mL, respectively) was detected, compared with the control (54.8 ± 1.6 pg/mL) (P < 0.05; Figure 5).

### GA-induced up-regulation of CSF-1R and tyrosine-phosphorylation of CSF-1R by activated microglia

**Figure 6:**
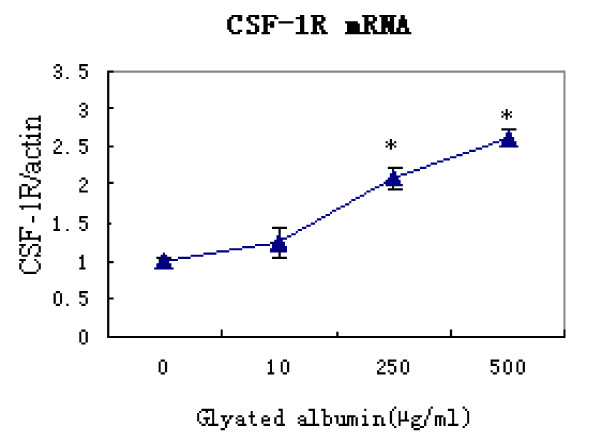
**Glycated albumin induces the up-regulation of CSF-1R mRNA expression in purified microglial cells**. Microglial cells were incubated with or without GA at indicated concentrations for 24 h, up-regulation of CSF-1R mRNA expression in microglia was observed. One-way ANOVA was performed, followed by post-hoc analysis. *, P < 0.0001.

Previous studies showed that CSF-1R expression is enhanced in activated microglia and that this up-regulation not only induces the microglial production of proinflammatory cytokines but also promotes the phagocytosis of amyloid β (Aβ). We analyzed the effect of GA on CSF-1R expression in microglia. CSF-1R mRNA was detected in unstimulated microglia; however, after 24 h of stimulation with 10, 250, or 500 μg/mL GA, the level of CSF-1R mRNA expression was increased 1.2-, 2.1-, and 2.6-fold, respectively (P < 0.05; Figure 6).

**Figure 7:**
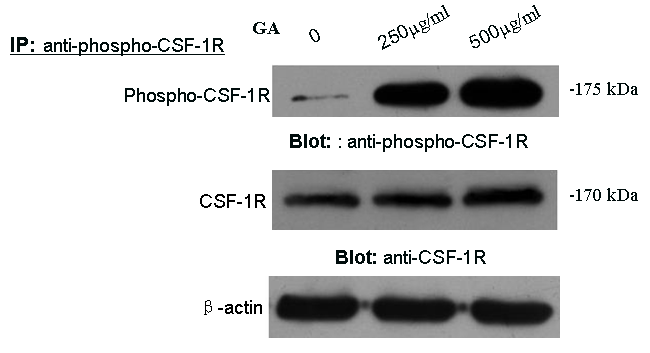
**Glycated albumin (GA) induces the up-regulation of CSF-1R and tyrosine-phosphorylation of CSF-1R in purified microglial cells**. Microglial cells were incubated with or without GA for 24 h and then subjected to immunoprecipitation (IP) with antibodies against phospho-CSF-1R(Tyr723) and then blot with antibodies against phospho-CSF-1R(Tyr723) or CSF-1R. The expression of β-actin was used as loading control.

To examine the effect of GA on CSF-1R signaling, we incubated microglial cells with 0, 250, or 500 μg/mL GA for 24 h, and then subjected them to immunoprecipitation and immunoblot analysis. Our results indicate that both CSF-1R and phosphorylation of CSF-1R (tyr273) was up-regulated by GA-activated microglia (Figure 7).

**Figure 8:**
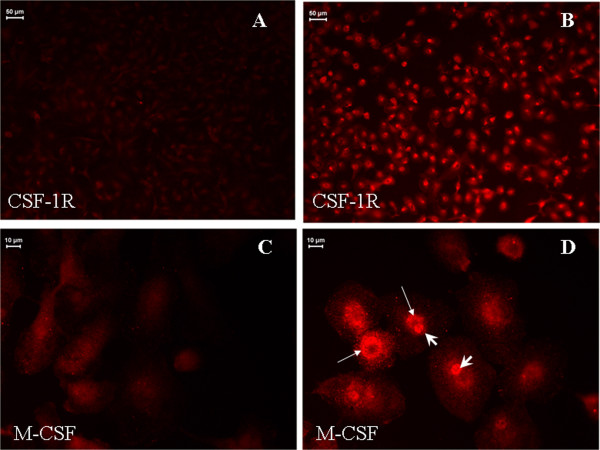
**Effect of glycated albumin (GA) on microglial CSF-1R and M-CSF expression**. (A) Without GA exposure, microglial cells exhibited weak CSF-1R immunofluorescence in the cytoplasm. (B) Following exposure to 250 μg/mL GA for 24 h, the expression of CSF-1R was clearly up-regulated. (C) Similarly, weak M-CSF immunofluorescence was observed in the cytoplasm without GA treatment. (D) Following exposure to 250 μg/mL GA for 24 h, the expression of M-CSF was up-regulated; the signal was detected in both the cytoplasm (long arrow) and nucleus (short arrow) of each cell.

Immunocytochemical analysis further demonstrated that GA induces the expression of CSF-1R (Figure 8A, 8B), as well as M-CSF (Figure 8C, 8D), in activated microglia. As shown in Figure 8, enhanced staining was observed in the GA-stimulated round amoeboid microglia, whereas weak staining was detected in the unstimulated ramified microglia.

### Inhibition of M-CSF or CSF-1R signaling suppresses GA-induced microglial inflammation

**Figure 9:**
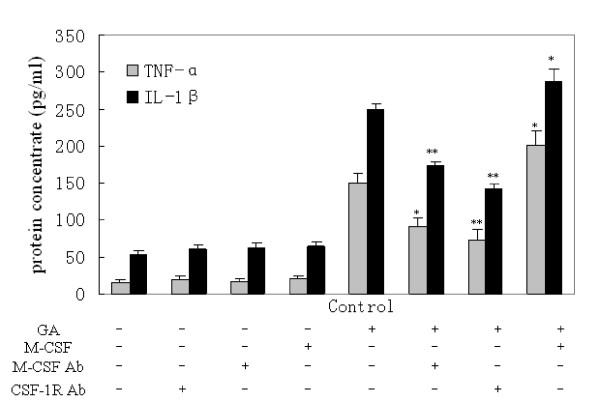
**Release of TNF-α and IL-1β from microglia following the neutralization of M-CSF or CSF-1R, or the administration of M-CSF**. None of the above had a significant effect on cytokine release in the groups without glycated albumin (GA) stimulation. The neutralization of M-CSF (1 μg/mL) or CSF-1R (1 μg/mL) suppressed TNF-α and IL-1β release in the experimental group (cells treated with 250 μg/mL GA for 24 h). Treatment with 100ng/mL M-CSF augmented the GA-induced release of TNF-α and IL-1β. One-way ANOVA was performed, followed by post-hoc analysis. *, P < 0.05; **, P < 0.001, compared with the control group.

We next tested our hypothesis that M-CSF/CSF-1R signaling promotes the release of proinflammatory cytokines in GA-stimulated microglia. We neutralized the activity of M-CSF or CSF-1R by using anti-M-CSF or anti-CSF-1R antibodies. The neutralization of M-CSF or CSF-1R had no effect on unstimulated microglia; however, both antibodies decreased the release of TNF-α and IL-1β from the GA-stimulated microglia (Figure 9).

### M-CSF augments GA-induced microglial inflammation

To further understand the combined effects of M-CSF and GA on the proinflammatory response in microglial cells, we included M-CSF in the microglial culture medium. M-CSF alone did not induce cytokine release; however, costimulation with M-CSF and GA for 24 h produced a significant increase in the release of TNF-□ and IL-1β (P < 0.05; Figure 9).

## Discussion

Inflammation involving vessels and neural tissue occurs early in diabetic retinopathy, while excessive and persistent microglial activation is believed a major contributor to the inflammatory responses [2,36,37]. Microglial activation occurs via a complex regulatory system involving multiple signals; however, the mechanism remains to be determined.

Amadori-glycated proteins and the early nonenzymatic glycation of proteins are increased in diabetes and have been independently associated with microvascular complications [14,15,38]. As the breakdown of the blood-retinal barrier (BRB) is a characteristic early event, the increased GA in plasma may penetrate from the compromised BRB and accumulate in retinal parenchyma, influencing the types of retinal cells and molecular mediators that subsequently promoting the development of diabetic retinopathy [17,18,39,40]. Our data suggested that *in vitro *GA activate microglia, producing proinflammatory factors. This event may be expected to occur in *vivo*, thereby aggravate the pathological inflammatory process. It was supported by the recent investigation that inhibiting the formation of GA would ameliorate the development of diabetic retinopathy [15].

The GA used in this study was produced commercially less than 1 week prior and purified to exclude residual contamination with AGEs as described by Baynes et al [41]. In contrast to AGEs, only a few studies have emphasized the role of GA, despite the fact that the Amadori product is a major form of glycated proteins and the concentration of GA exceeds that of AGEs [14,42]. Amadori-modification is structurally distinct from AGEs, and can bind to monocytes/macrophages via specific receptors [19,20]. These receptor proteins have been characterized and the amino acid sequence homologies were not found in any of the AGE receptors [19-21,43]. Therefore, it is reasonable that binding of GA to microglia via specific receptors rather than receptors of AGEs, induced microglial activation.

Accumulating evidences showed M-CSF is a key cytokine in the regulation of the microglial activation, proliferation and migration in CNS [23-25]. Moreover, the high-affinity binding of M-CSF to CSF-1R plays a role in mononuclear/macrophage-associated diabetic complications, including diabetic retinopathy [32,33,44,45]. Our previous study provided the evidence that the vigorous expression of M-CSF/CSF-1R occurred in the early diabetic retina and a robust induction of CSF-1R was observed on the activated microglia [46]. Furthermore, as shown in the current study, primary retinal microglial cells express either CSF-1R or M-CSF, albeit at relatively low baseline levels. While they were exposed to gradient concentrations of GA, the enhanced expression of CSF-1R signaling was detected in activated microglia, not only at the mRNA but also at the protein level. This up-regulation is consistent with that as seen *in vivo*, and may be integral to diabetic microglial activation [46]. In light of the above findings, we hypothesize that M-CSF signaling is a possible molecular pathway in diabetic retinopathy.

To further our understanding, we measured the amounts of TNF-α and IL-1β, two important proflammatory cytokines secreted by activated microglia, in GA-stimulated microglial cultures treated with or without additional M-CSF. Our results show that M-CSF enhances the production of proflammatory cytokines in GA-activated microglia. M-CSF alone, however, did not induce the proinflammatory responses but proliferate microglia as shown by MTT assay. Although theoretically, more cells due to M-CSF treatment may contribute to more cytokine production, given the fact that this inducible proinflammation could be significantly inhibited by antibody neutralization of M-CSF or CSF-1R, and the fact that GA enhanced expression of M-CSF/CSF-1R by microglia, it is reasonable that this is quite far from the actual synergistic effects of the two agents. Taken all together, we concluded that M-CSF may be a co-stimulator with GA and that M-CSF/CSF-1R signaling exerts a synergistic effect with GA on the production of proinflammatory cytokines, not simply due to effects on cell proliferation.

Astrocytes, a major source of M-CSF in CNS, can be induced to release M-CSF by proinflammatory cytokines [47-50]. However, note that M-CSF is also secreted by activated microglial cells [26,28]. Moreover, as determined by immunocytochemistry and ELISA, the level of M-CSF elevates in GA-stimulated-microglia. Elevated M-CSF expression could cause further reactive microgliosis, phagocytosis, and the release of inflammatory cytokines such as IL-1β, IL-6, and M-CSF [23-25]. Thus, the present study provides evidence for the roles of M-CSF and its signaling cascade in activated microglia in response to GA stimulation, suggesting that M-CSF is an important cross-talk mediator, involved in astrocytes, neurons, and microglia.

## Conclusions

In summary, it is evident that GA can induce microglial activation by stimulating the release of proinflammatory cytokines such as TNF-α and IL-1β *in vitro*. Simultaneously, up-regulation of CSF-1R on microglia may augment the overall inflammatory response by propagating the proinflammatory signal to nearby resting microglia and astrocytes. The binding of M-CSF to overexpressed CSF-1R seems to augment GA-induced microglial inflammation via autocrine and paracrine effects, which can be blocked by antibody neutralization. Our present study demonstrate that M-CSF/CSF-1R signaling represents a further link between microglial inflammation and diabetic retinopathy and raises the possibility of specific therapies for targeting microglia-mediated injury in diabetic retinopathy. Further work are required for a better understanding of the molecular mechanisms underlying M-CSF/CSF-1R signaling in microglia activation.

## Methods

### Primary Retinal Microglial Culture

Primary mixed glial cell cultures were prepared as described previously with modifications [51,52]. In brief, fresh retinas were obtained from newborn Sprague-Dawley rats (2-3 days old). Animal care procedures and the experimental protocol complied with the Guidelines of the Ethics Committee at Fudan University. The retinas were placed on ice in Ca^2+^- and Mg^2+^-free Hank's balanced salt solution after removal of the blood vessels. The tissues were then minced and exposed to 0.25% trypsin-EDTA (Invitrogen, Carlsbad, CA) at 37°C for 8 min. After terminating the digestion with fetal bovine serum (FBS, Invitrogen), the cells were collected by centrifugation, re-suspended in Dulbecco's modified Eagle's medium (DMEM)/F12 (1:1) (Invitrogen) supplemented with 10% FBS, penicillin/streptomycin (100 U/100 μg per mL), and L-glutamine (2 mM), and plated on poly L-lysine-coated (Sigma-Aldrich, St. Louis, MO) T75 flasks at a density of 1.2 × 10^6 ^cells/mL. The culture medium was changed after 3 days and then twice a week. Upon confluence (7-10 days), the microglial cells were harvested by the "shaking-off" method (an initial shake at 100 rpm for 30 min followed by a second shake 7 days later). The purified microglial cells were then reseeded in 24-well plates or T25 flasks (Corning, Inc., Corning, NY). The purity of the cells was assessed by immunocytochemistry using the monoclonal antibody OX-42 (a microglial marker for complement type 3 receptor) and anti-glial fibrillary acidic protein antibodies (GFAP) (an astrocyte marker). Cellular morphology was examined under a phase-contrast microscope.

### Assessment of viabilities by MTT assay

For MTT assay, microglia (2.5 × 10^4 ^cells in 200 μl/well) were seeded in 96-well plates for 24 h and treated with various stimuli for 48 h. After the treatment, the medium was removed and MTT (5 mg/mL, 20 μl/well) was added, followed by incubation at 37°C for 4 h. Afterward, supernatants were carefully removed and DMSO (150 μl/well, Sigma-Aldrich, St. Louis, MO) was added to the cells. After insoluble crystals were completely dissolved, absorbance at 490 nm was measured using Thermomax microplate reader (Molecular Devices).

### GA-Induced Retinal Microglial Activation

Purified microglial cells were seeded into T25 flasks. After 48 h of growth, the cells were incubated with serum-free DMEM/F-12 containing 0, 10, 50, 100, or 250 μg/mL human GA for 24 h. The GA used in this study (glycated human serum albumin from Sigma-Aldrich) was made less than 1 week prior as described by Baynes et al. [41], and contained 2.7-3.5 mol of fructoselysine per mol of albumin. No measurable AGEs were detected in these products by fluorescence assay (from 360 to 600 nm) with excitation at 370 or 350 nm, or by Western blot analysis [18,53,54]. To rule out the possible effects of lipopolysaccharide (LPS) contamination as a potential activator of microglia, microglial cells were also incubated with 250 μg/mL GA for 24 h in the absence or presence of 10 μg/mL PMXB (Sigma-Aldrich). Each experiment was performed in triplicate.

### M-CSF Treatment

Recombinant murine M-CSF (R&D Systems, Minneapolis, MN) was added at a concentration of 50 ng/mL to the cell medium with or without 250 μg/mL human GA. The cells were then allowed to grow for 24 h.

### M-CSF and CSF-1R Neutralization

For neutralization, 1 μg/mL anti-M-CSF or anti-CSF-1R antibodies (Santa Cruz Biotechnology, Santa Cruz, CA) were added to the cell medium. After 2 h of preincubation at 37°C, an additional 250 μg/mL human GA were added to the medium.

### Immunocytochemistry

For immunocytochemistry, 12-mm glass coverslips coated with poly L-lysine were used. Cells were fixed with 4% paraformaldehyde in 0.1 M phosphate buffer for 15 min at room temperature, washed in 0.01 M phosphate-buffered saline (PBS, pH 7.4), blocked in 5% bovine serum for 30 min, and incubated overnight with the primary antibodies. The following antibodies were used: OX-42 (1:200 dilution; Chemicon, Temecula, CA), Iba1 (1:200; Abcam, Cambridge, UK), M-CSF (1:100; Santa Cruz Biotechnology), and CSF-1R (1:100; Santa Cruz Biotechnology). After three rinses with 0.01 M PBS, fluorescein isothiocyanate (FITC)-conjugated anti-mouse antibodies (1:200; Sigma-Aldrich), tetramethyl rhodamine isothiocyanate (TRITC)-conjugated anti-goat antibodies (1:200; KPL), or Cy3/IgG-conjugated anti-rabbit antibodies (1:200; Sigma-Aldrich) were added as secondary antibodies, and the cells were incubated at 37°C for 1 h. To visualize the nuclei, 4', 6-diamidino-2-phenylindole (DAPI, 1:500; Sigma-Aldrich) was used. Images were acquired using a fluorescence microscope or laser confocal microscope (TCS SP2; Leica Microsystems, Mannheim, Germany). The optical sections were reconstructed with a maximum projection using the software provided with the microscope (Leica Microsystems).

### Real-Time PCR

Purified microglial cells were starved of serum for 6 h and then restimulated with graded GA for 24 h. The microglia was then sorted into RNA lysis buffer, frozen on dry ice, and kept at -80°C until processing. Total RNA was isolated with Trizol reagent (Invitrogen). The quality and quantity of the RNA were determined using a biophotometer (DU 800; Beckman Coulter, Fullerton, CA).

Rat retinal cDNA was prepared from 2 μg of total RNA using SuperScript™ reverse transcriptase (RT; Life Technologies, Grand Island, NY). Briefly, isolated RNA was incubated with 50 μM oligo (dT) 20, 10 mM dNTP mix, and DEPC-treated water at 65°C for 5 min. Next, 10 μl of cDNA synthesis mix (10 × RT buffer, 25 mM MgCl_2_, 0.1 M DTT, 40 U/μl RNaseOUT, and 200 U/μl SuperScript™ III RT) were added to the reaction, followed by incubation with oligo (dT) 20 at 50°C for 50 min, then incubation with random hexamer at 25°C for 10 min, and finally incubation at 50°C for another 50 min. The reactions were terminated by treatment at 85°C for 5 min, and the mixtures were chilled. RNase H was added to each sample, and the mixture was incubated for 20 min at 37°C.

The synthesis of cDNA was achieved using the following: CSF-1R sense, 5'-GCCTTTGGTCTGGGCAAA-3'; CSF-1Rantisense, 5'-AGCCGTGGACTTGAGCATCT-3'; and TaqMan probe, 5'-FAM-AAGATGCAGTGCTGAAGGTGGCTGTG-TAMRA-3'. The primers and probe were designed using Primer Express software (ver2.0; PE Applied Biosystems, Foster City, CA). Quantitative PCR amplification was carried out on a real-time PCR machine (Applied Biosystems 7500 real-time PCR system), using an initial incubation for 2 min at 95°C followed by 40 cycles of 15 s at 95°C and 45 s at 60°C. The mRNA level of CSF-1R was standardized against the β-actin mRNA level in the same RNA sample. Each experiment was performed three times.

### Immunoprecipitation and Western Blotting

Cells were collected and lysed in lysis buffer [50mM Tris-HCl, pH 7.5, 1mM EDTA, 0.1% SDS, 150 mM NaCl, 5 mM NaF, 0.5% sodium deoxycholate, 1% Nonidet P-40, and 1% protease inhibitor cocktail (Sigma-Aldrich)]. The lysates were centrifuged at 14,000 × *g *for 10 min at 4°C and precleared by incubation with 25 μl of protein A/G-Sepharose beads (GE Healthcare, Piscataway, NJ) and 2 μg of normal murine serum at 4°C for 1 h. The precleared lysates were then mixed with 5 μg of anti-CSF-1R antibodies and 20 μl of protein A/G-Sepharose beads as described above and incubated overnight with gentle rocking at 4°C. The beads were isolated by centrifugation and washed four times with cell lysis solution. The bound proteins were subjected to 8% SDS-PAGE and transferred to a polyvinylidene difluoride (PVDF) membrane (Millipore, Billerica, MA). After incubation for 1 h in blocking solution (5% nonfat dry milk in 0.1% TBST buffer), the membrane was incubated overnight at 4°C in blocking buffer containing the respective antibody (anti-phospho-CSF-1R(Tyr723),1:200; Cell Signaling Technology; anti-CSF-1R, 1:200, Santa Cruz Biotechnology). The membrane was then washed three times and incubated with horseradish peroxidase-conjugated goat anti-rabbit antibodies (1:2,000; Santa Cruz Biotechnology) in blocking buffer for 2 h at room temperature. The bound antibodies were detected using an enhanced chemiluminescence system (ECL; GE Healthcare) and X-ray film. Densitometric analysis of each sample was performed using ImageMasterRVDS (GE Healthcare).

### Enzyme-Linked Immunosorbent Assay (ELISA)

Purified microglial cells were incubated overnight in 24-well plates at 2.5 × 10^5 ^cells per well in 1 mL of serum-free medium with or without graded GA. The supernatant was collected at the indicated times after treatment with the conditioned medium from the control and experimental groups, and the amounts of TNF-α, IL-1β, and M-CSF were quantified by ELISAs (Biosource, Inc., Camarillo, CA), in accordance with the manufacturer's instructions. Each experiment was performed three times.

### Statistical Analysis

Statistical analyses were performed using one-way ANOVA, followed by post-hoc analysis with the Bonferroni (equal variances assumed) or Dunnett's test (equal variances not assumed). All quantitative data are presented as the mean ± SD. Values of P < 0.05 were considered statistically significant.

## Authors' contributions

LW performed the experiments, designed the protocol, performed the statistical analysis and drafted the manuscript. XGZ and JCH designed the protocol and coordinated the study. TJ participated in the microglial culture. All authors have read and approved the final manuscript.

## Competing interests

The authors declare that they have no competing interests.
